# Supplementary material for: Broadband perfect light trapping in the thinnest monolayer graphene-MoS2 photovoltaic cell: the new application of spectrum-splitting structure
Source: Sci Rep. 2016 Feb 11;6:20955. doi: 10.1038/srep20955 (PMC4750090; doi:10.1038/srep20955)
Supplement: Supplementary Information [file srep20955-s1.doc]

Supplementary Materials:

Broadband perfect light trapping in the thinnest monolayer graphene-MoS_2_ photovoltaic cell: the new application of spectrum-splitting structure

Yun-Ben Wu^1^, Wen Yang^2^, Tong-Biao Wang^3^, Xin-Hua Deng^3^, Jiang-Tao Liu^1,3*^

^1^Nanoscale Science and Technology Laboratory, Institute for Advanced Study, Nanchang University, Nanchang 330031, China

^2^Beijing Computational Science Research Center, Beijing 100084, China

^3^Department of Physics, Nanchang University, Nanchang 330031, China

Corresponding Author Email: [jtliu@semi.ac.cn](mailto:jtliu@semi.ac.cn)

The supplement includes more details on:

S1. Transfer matrix method.

S2. Distributed Bragg reflector with parallel layers.

S3. The influence of the fabrication tolerances of the photonic crystal.

S4. The outermost layer correction.

S5. The tolerances of the photovoltaic cells position.

S6. The external quantum efficiency of the proposed GM-PV cell.

**S1. Transfer matrix method.**

The modified transfer matrix method is used to model the absorption of monolayer graphene-MoS2 photovoltaic cell (GM-PV cell) in the photonic crystal microcavity. In the *l*-th layer, the electric field and the magnetic field of the TE mode light beam with incident angle $\theta_{i}$is given by [R1]

$\left\{\begin{matrix} \mathbf{E}_{l}(x,y)=[A_{l}e^{-k_{lix}(x-x_{l})}e^{ik_{lrx}(x-x_{l})}+B_{l}e^{k_{lix}(x-x_{l})}e^{-ik_{lrx}(x-x_{l})}]e^{-k_{liy}y}e^{ik_{lry}y}\mathbf{e}_{\text{z}}, \\ \mathbf{H}_{l}(x,y)=\frac{1}{\omega\mu_{0}\mu_{l}}\left(k_{ly}\mathbf{e}_{\text{x}}-k_{lx}\mathbf{e}_{\text{y}}\right)[A_{l}e^{-k_{lix}(x-x_{l})}e^{ik_{lrx}(x-x_{l})}-B_{l}e^{k_{lix}(x-x_{l})}e^{-ik_{lrx}(x-x_{l})}]e^{-k_{liy}y}e^{ik_{lry}y}\text{, } \end{matrix}\right.\text{ (1)}$where $k_{l}=k_{lr}+ik_{li}$ is the wave vector of the THz wave, $\text{(}\mathbf{e}_{\text{x}},\mathbf{e}_{\text{y}},\mathbf{e}_{\text{z}})$ is the unit vectors in the (x,y,z)-direction respectively, and $x_{l}$is the position of the *l*-th layer in the x-direction, $\omega$ is the circular frequency of the light beams, $\mu_{0}$is the permeability of vacuum, $\mu_{l}=1$is the permeability of the *l*-th layer. And the electric field and magnetic field of the TM mode light beam with incident angle $\theta_{i}$is given by

$$\left\{\begin{matrix} \mathbf{H}_{l}(x,y)=[A_{l}e^{-k_{lix}(x-x_{l})}e^{ik_{lrx}(x-x_{l})}+B_{l}e^{k_{lix}(x-x_{l})}e^{-ik_{lrx}(x-x_{l})}]e^{-k_{liy}y}e^{ik_{lry}y}\mathbf{e}_{\text{z}}, \\ \mathbf{E}_{l}(x,y)=\frac{-1}{\omega\varepsilon_{0}\varepsilon_{l}}\left(k_{ly}\mathbf{e}_{\text{x}}-k_{lx}\mathbf{e}_{\text{y}}\right)[A_{l}e^{-k_{lix}(x-x_{l})}e^{ik_{lrx}(x-x_{l})}-B_{l}e^{k_{lix}(x-x_{l})}e^{-ik_{lrx}(x-x_{l})}]e^{-k_{liy}y}e^{ik_{lry}y}\text{, } \end{matrix}\right.\text{ (2)}$$

the electric fields of TE mode or the magnetic fields of TM mode in the (*l*+1)-th and *l*-th layer are related by the matrix utilizing the boundary condition

$$\left(\begin{matrix} A_{l+1} \\ B_{l+1} \end{matrix}\right)=\left(\begin{matrix} \frac{\gamma_{l}+\gamma_{l+1}}{2\gamma_{l+1}}e^{-k_{lix}d_{l}}e^{ik_{lx}d_{l}}\begin{matrix} & \frac{\gamma_{l+1}-\gamma_{l}}{2\gamma_{l+1}}e^{k_{lix}d_{l}}e^{-ik_{lx}d_{l}} \end{matrix} \\ \frac{\gamma_{l+1}-\gamma_{l}}{2\gamma_{l+1}}e^{-k_{lix}d_{l}}e^{ik_{lx}d_{l}}\begin{matrix} & \frac{\gamma_{l}+\gamma_{l+1}}{2\gamma_{l+1}}e^{k_{lix}d_{l}}e^{-ik_{lx}d_{l}} \end{matrix} \end{matrix}\right)\left(\begin{matrix} A_{l} \\ B_{l} \end{matrix}\right)=\left(\begin{matrix} t_{11}^{l} & t_{12}^{l} \\ t_{21}^{l} & t_{22}^{l} \end{matrix}\right)\left(\begin{matrix} A_{l} \\ B_{l} \end{matrix}\right)\text{, (3)}$$

where $\gamma_{l}=\frac{k_{lrx}+ik_{lix}}{\omega\mu_{0}\mu_{l}}$ ($\gamma_{l}=\frac{k_{lrx}+ik_{lix}}{\omega\varepsilon_{0}\varepsilon_{l}}$) for TE (TM) mode, $d_{l}$ is the thickness of the *l*-th layer. The light in the *l*-th layer is related to the incident fields by the transfer matrix

$$\left(\begin{matrix} A_{l} \\ B_{l} \end{matrix}\right)=\left(\begin{matrix} t_{11}^{l-1} & t_{12}^{l-1} \\ t_{21}^{l-1} & t_{22}^{l-1} \end{matrix}\right)\cdots\left(\begin{matrix} t_{11}^{0} & t_{12}^{0} \\ t_{21}^{0} & t_{22}^{0} \end{matrix}\right)\left(\begin{matrix} A_{0} \\ B_{0} \end{matrix}\right)=\left(\begin{matrix} T_{11} & T_{12} \\ T_{21} & T_{22} \end{matrix}\right)\left(\begin{matrix} A_{0} \\ B_{0} \end{matrix}\right)\text{. (4)}$$

Thus, we can obtain the absorptance of the *l*-th layer to (*l+m*)-th layer $\zeta_{a}$ using the Poynting vector $\mathbf{S}=\mathbf{E}\times\mathbf{H}$

$$\zeta_{a}=[\mathbf{S}_{(l-1)i}+\mathbf{S}_{(l+m+1)i}-\mathbf{S}_{(l-1)o}-\mathbf{S}_{(l+m+1)o}]/\mathbf{S}_{0i}\text{, (5)}$$

where $\mathbf{S}_{(l-1)i}$ and $\mathbf{S}_{(l-1)o}$ [$\mathbf{S}_{(l+m+1)i}$ and $\mathbf{S}_{(l+m+1)o}$] are the incident and outgoing Poynting vectors in (*l-1*)-th and [(*l+m+1*)-th] layers, respectively, and $\mathbf{S}_{0i}$ is the incident Poynting vector in air.

**S2. Distributed Bragg reflector with parallel layers**

Fig. S1. Absorptance of shoulder-to-shoulder GM-PV subcells with different thickness planar-shaped DBR (inset: schematic of shoulder-to-shoulder GM-PV subcells).


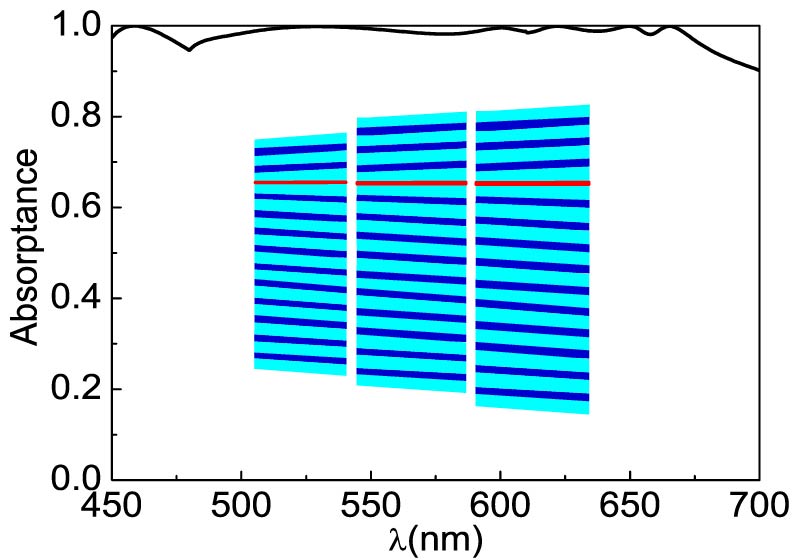


To expand the photonic band gap of the distributed Bragg reflector (DBR), wedge-shaped DBR is used in the calculation. Usually, the DBR is fabricated with planar-shaped layers. Limited to the photonic band gap, perfect absorption is difficult to achieve in a wide wavelength range. But by using shoulder-to-shoulder subcells with different DBR, nearly perfect absorption can be achieved over a broad spectrum range [Fig. S1].

**S3. The influence of the fabrication tolerances of the photonic crystal.**

Fig. S2. Absorptance of GM-PV cell when a random thickness variation is introduced in each layer in DBR, standard deviation (a)
 nm, (b)
 nm, and (c)
 nm


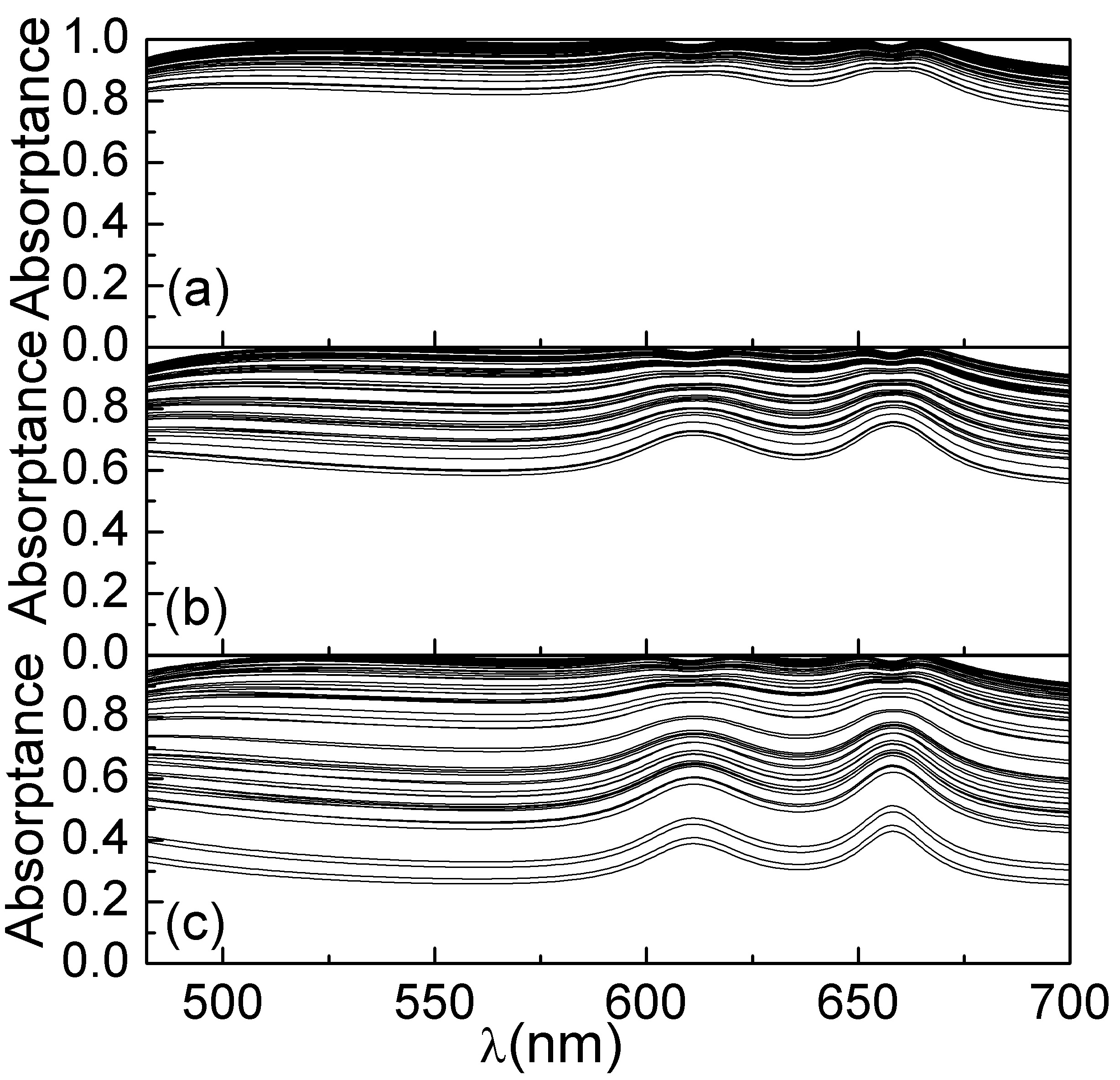


The thickness variations of the layers in DBR cause the reflectivity and the resonant frequency of the cavity may change as well. Similar to the actual fabrication process, random thickness variation is introduced in each layer in DBR. The thickness variation $\Delta d_{l}$ follows a normal distribution $f\left(\zeta\right)=\frac{1}{\sqrt{2\pi}\bullet\delta}\exp[-\frac{\zeta^{2}}{2\delta^{2}}]$, where parameter $\delta$ is the standard deviation. The normal distribution is simulated by using the Box–Muller transform [R2]. The calculated results are illustrated in Fig. S2. In each Figure, the absorptance is calculated by 60 times with random thickness variation. For $\delta=1$ nm, more than 91.7% of the results show that the average absorptance is larger than 0.9. For $\delta=2$ nm ($\delta=3$ nm), more than 55% (43%) percent of the results show that the average absorptance is larger than 0.9 and more than 70% (62%) percent of the results show that the average absorptance is larger than 0.8. For $\delta=2$ nm ($\delta=3$ nm), the lowest average absorptance is only about 0.65 (0.35), which is caused by the large thickness variation (>5 nm) in the layer most close to the defect layer.

**S4. The outermost layer correction.**

Fig. S3. Absorptance of GM-PV cell with defect layer thicknesses variation
 and outermost layer thicknesses correction
. The inset shows the perfect matched correction (blue dash-dotted line) and wedge-shaped correction (red dotted line).


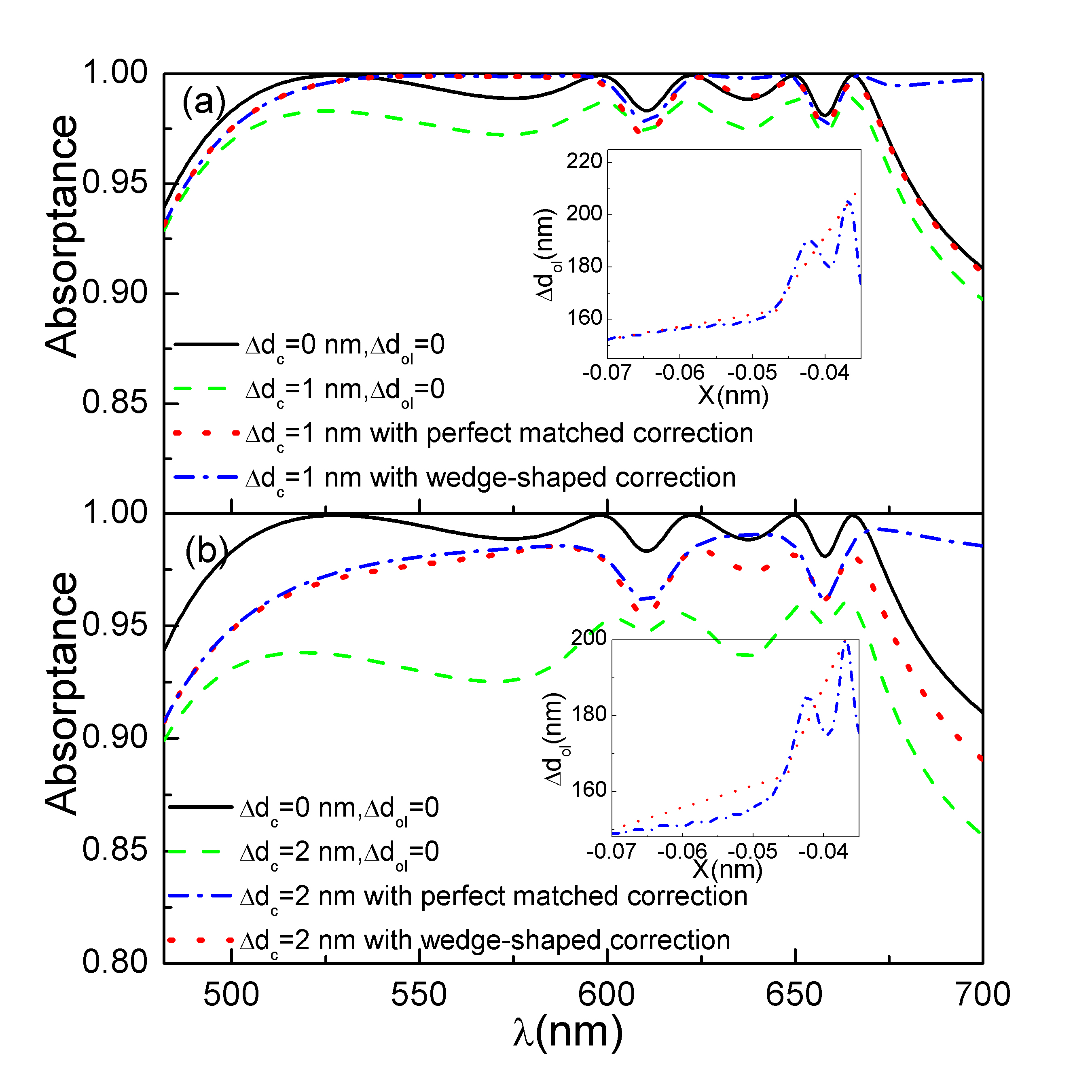


As an interference effect, the cavity resonant frequency depends not only on the optical path length of the defect layers but also on the total optical path length of the DBR. Thus, by adding a thickness correction $\Delta d_{ol}$ in the outermost layer, the resonant frequency of the cavity can be adjusted. The reflectivity of the upper DBR can also be adjusted by varying the outermost layer thickness, which will lead to variation of the Q-value of the microcavity. Numerical results are shown in Fig. S3. By adjusting the defect layer thickness, a resonance-induced absorption can be achieved (i.e., $\Delta d_{c}=0$, $\Delta d_{ol}=0$, black sold line). If the defect layer thickness reduces $\Delta d_{c}$, the resonance-induced perfect absorption of the light incident on the cavity can no longer be achieved. The absorptance is reduced ($\Delta d_{c}=1$nm or $\Delta d_{c}=2$nm, $\Delta d_{ol}=0$, green dashed line). However, when introducing the outermost layer perfect matched correction (bule dash-dotted)or wedge-shaped correction (red dotted line), the absorption can be enhanced. Especially, a maximum absorption around $\lambda=580$ nm can be achieved even with $\Delta d_{c}=1$ nm by introducing the correction. It is because that the Q-value of the microcavity can be adjusted by varying the thickness of the outermost layer. Therefore, even if the manufacturing deviation occurs, by adjusting the thickness of the outermost layer it is possible to improve the quality and reduce the existing number of discarded products.

**S5. The tolerances of the photovoltaic cells position.**

When the x position of the GM-PV cell has a certain deviation, the absorptance of GM-PV cell decreases due to the lack of perfect matching. This variation is equivalent to the variation in the thickness of the defect layer. The slope of the defect layer thickness variation is approximately $80nm/60mm\approx1.33\times10^{-6}$. The variation of 1.5 mm in the x position means that the defect layer thickness exhibits a change of 2 nm, i.e., the deviation of x position impose significant effects on the absorptance of GM-PV cell. Certainly, with more efficient spectrum-splitting system, the slope of thickness variation can be reduced and thus the effects induced by the deviation of x-position can be weakened. Additionally, the adjustment can be implemented with a feedback mechanical system.

The deviation of the GM-PV cell z-position is equivalent to the change of spot size. Its effect is equivalent to that of aberration. Smaller diameter of the lens and longer focal length means less effect induced by the deviation of z-position. When the diameter is 10 cm and the focal length is 50 cm, if the deviation of z-postion is 0.5 cm, the diameter of the light spot will increase by about 1 mm, smaller than the variation of light spot induced by aberration, i.e., the effect is little.

**S6. The external quantum efficiency of the proposed GM-PV cell.**

Finally, we discuss the external quantum efficiency of the proposed GM-PV cell. As shown in the main tex of the paper, the GM-PV cell exhibits up to >90% light absorptance in the 450-670 nm wavelength range. By choose appropriate materials of DBR and spectrum-splitting structure, broadband perfect absorption can be achieved in <450 nm wavelength range. Thus, >25% of the total power spectrum from the sun can be absorbed by the proposed GM-PV cell. The external quantum efficiency can be larger than 20%. To get perfect absorption with wavelength >700 nm, shoulder-to-shoulder cells consisting of narrower band gap graphene-MoSe_2_ (1.5 eV) and graphene-MoTe_2_ (1.1 eV) [R5] should be added [R6]. The estimated external quantum efficiency can be larger than 80%. As a comparison, we also show the absorption of the GM-PV cell in Ref. [R3] in Fig. S4. By using the chirped-planar-dielectric cavities, the absorption of GM-PV cell can absorb as much as 33% of incident visible light over a 300 nm bandwidth, and the external quantum efficiency of GM-PV cell can be enhanced 3.6 times to a predicted value of 7.09%.

Fig. S4. Absorption of the GM-PV cell in this paper (red dashed line) and in Ref. [R3] (black solid line). The inset shows the AM1.5G spectrum[R4]


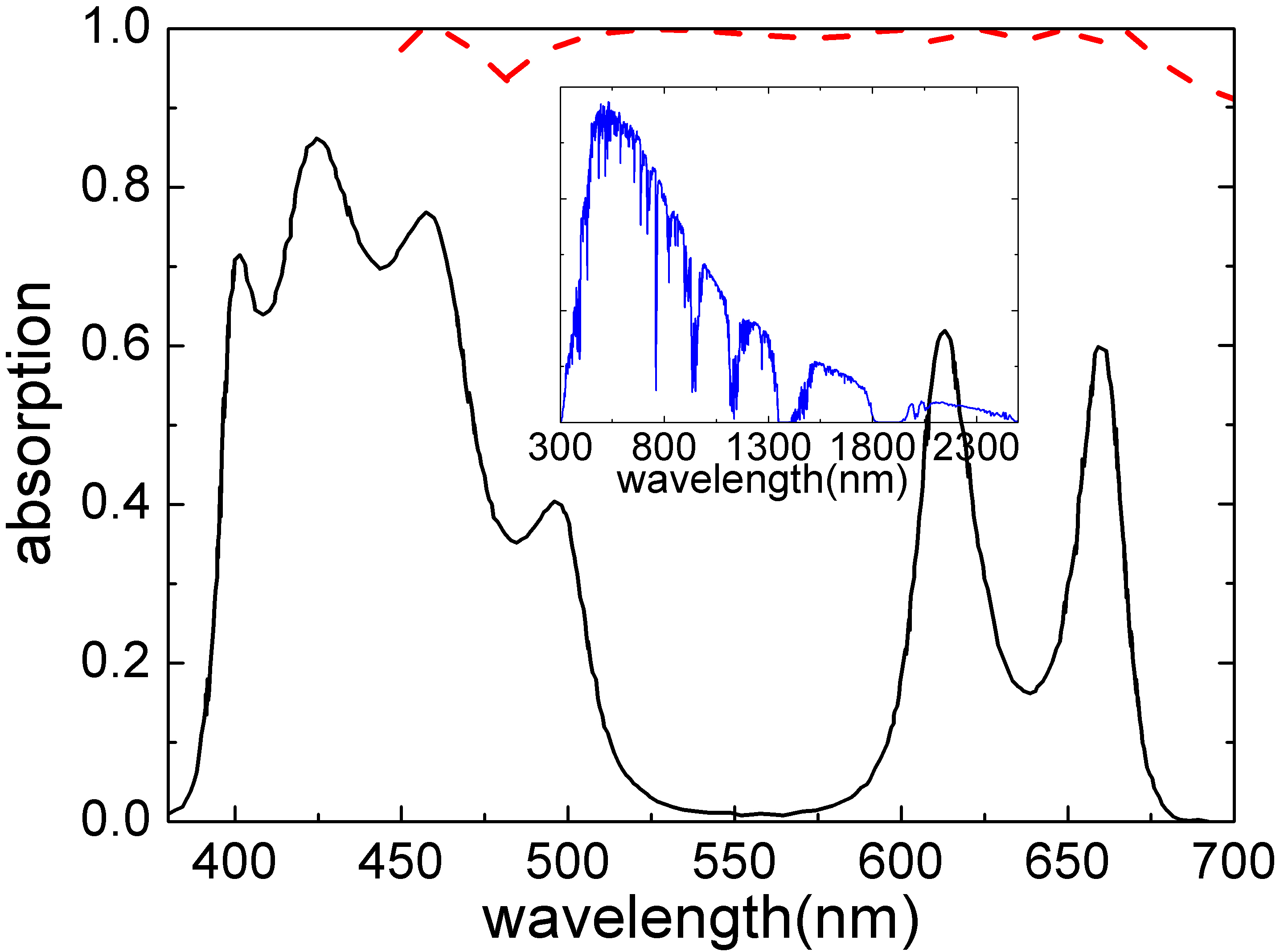


**Referrence**

[R1] Born and E. Wolf, Principles of Optics. Pergamon, Oxford, U.K., (1989), pp. 38–74.

[R2] G. E. P. Box and Mervin E. Muller, A Note on the Generation of Random Normal Deviates, The Annals of Mathematical Statistics (1958), Vol. 29, No. 2 pp. 610–611.

[R3]The AM1.5G spectrum was taken from the NREL website: http://rredc.nrel.gov/solar/spectra/am1.5 and integrated with the trapezoid rule.

[R5] Wang, Q. H., Kalantar-Zadeh, K., Kis, A., Coleman, J. N. & Strano, M. S. Electronics and optoelectronics of two-dimensional transition metal dichalcogenides. Nat. Nanotech. 7, 699–712 (2012).

[R6]Liu, J.-T., Deng, X.-H., Yang, W. & Li, J. Perfect light trapping in nanoscale thickness semiconductor films with a resonant back reflector and spectrum-splitting structures. Phys. Chem. Chem. Phys. 17, 3303 (2015).
